# Myosin II Motor Proteins with Different Functions Determine the Fate of Lamellipodia Extension during Cell Spreading

**DOI:** 10.1371/journal.pone.0008560

**Published:** 2010-01-05

**Authors:** Venkaiah Betapudi

**Affiliations:** 1 Department of Cell Biology, Lerner College of Medicine of Case Western Reserve University, Cleveland, Ohio, United States of America; 2 Department of Physiology and Biophysics, Cleveland Clinic Foundation, The Lerner Research Institute, Case Western Reserve University, Cleveland, Ohio, United States of America; Dresden University of Technology, Germany

## Abstract

Non-muscle cells express multiple myosin-II motor proteins myosin IIA, myosin IIB and myosin IIC transcribed from different loci in the human genome. Due to a significant homology in their sequences, these ubiquitously expressed myosin II motor proteins are believed to have overlapping cellular functions, but the mechanistic details are not elucidated. The present study uncovered a mechanism that coordinates the distinctly localized myosin IIA and myosin IIB with unexpected opposite mechanical roles in maneuvering lamellipodia extension, a critical step in the initiation of cell invasion, spreading, and migration. Myosin IIB motor protein by localizing at the front drives lamellipodia extension during cell spreading. On the other hand, myosin IIA localizes next to myosin IIB and attenuates or retracts lamellipodia extension. Myosin IIA and IIB increase cell adhesion by regulating focal contacts formation in the spreading margins and central part of the spreading cell, respectively. Spreading cells expressing both myosin IIA and myosin IIB motor proteins display an organized actin network consisting of retrograde filaments, arcs and central filaments attached to focal contacts. This organized actin network especially arcs and focal contacts formation in the spreading margins were lost in myosin IIÂ cells. Surprisingly, myosin IIB̂ cells displayed long parallel actin filaments connected to focal contacts in the spreading margins. Thus, with different roles in the regulation of the actin network and focal contacts formation, both myosin IIA and IIB determine the fate of lamellipodia extension during cell spreading.

## Introduction

Cell migration plays a fundamental role in the development and maintenance of the normal physiology of every organism. Deregulation of cell migration is implicated in cancer spread, mental retardation, infection, and vascular diseases. Cells initiate migration by extending their plasma membrane in the form of lamellipodia that requires the orchestration of the cell cytoskeleton [1]. As a part of this dynamic process, monomeric G-actin polymerizes into filaments (F-actin) that undergo rearrangements and depolymerization during cell spreading and migration [2]–[9]. Nonmuscle myosin II, a conventional motor protein known to generate intracellular contractile forces and tension by associating with F-actin, has been implicated in driving cell spreading, migration, cytokinesis, and other cellular processes [10]–[13]. Most nonmuscle cells express myosin IIA, myosin IIB, and myosin IIC motor proteins. Each myosin II motor protein exists as a complex consisting of two copies each of myosin II heavy chain (MHC), essential light chains (ELC), and regulatory light chain (RLC). The MHCs of myosin IIA, IIB, and IIC motor protein complexes are encoded by *Myh9*, *Myh10*, and *Myh14* genes, respectively [14]–[16]. The MHC consists of an N-terminal globular motor domain having binding sites for ATP and F-actin, a neck region that binds to RLC and ELC, and a C-terminal α-helical coiled-coil tail domain. Myosin II motor proteins are ubiquitously expressed and display 64–89% similarity in the amino acid sequences of their heavy chains [17]. Due to such considerable homology in their amino acid sequences, these myosin II motor proteins are believed to have overlapping cellular functions. However, these myosin II motor proteins show difference in their motor activities, molecular interactions, cellular, and tissue distributions [18]–[26]. Myosin IIB is required for driving the outgrowth of the neuritic processes and the role of myosin IIA is implicated in mediating neurite retraction [27]–[30]. Myosin IIB is shown to mediate exocytosis, an essential cellular process known to secrete signaling molecules or other cellular products at the leading edge of migrating mammalian cells [31], [32]. Myosin IIB involves in vesicle trafficking to presynaptic terminals of cultured superior cervical ganglion neurons [32]. By directly interacting, myosin IIA mediates CXCR4 chemokine receptor endocytosis in migrating T lymphocytes [33]. Myosin IIA binds to Mts1, a member of the S100 family of Ca2+-binding proteins that is directly involved in tumor invasion and metastasis [34]. Myosin IIC, a newly discovered class II motor protein is believed to have roles in regulating cell membrane extension and focal contacts formation [35]. Recent studies from our laboratory showed opposite roles for myosin IIA and myosin IIB in extending lamellipodia, a critical step in the initiation of cell invasion, spreading and migration [36]. However, the underlying mechanism of lamellipodia extension driven by myosin IIA and IIB motor proteins is not clearly understood.

The present study is performed to understand the specific roles of myosin IIA and IIB in regulating focal contacts and actin network formation that are critical to lamellipodia extension. The present study reported distinct and opposite roles of myosin IIA and IIB motor proteins in driving lamellipodia extension during cell spreading.

## Materials and Methods

### Cell Lines, Chemicals and Antibodies

The MDA-MB-231 human breast cancer cell line and HeLa cell line were obtained from the American Type Culture Collection (Rockville, MD). Tet regulated HeLa cell line was from Clontech (Mountain View, CA) and COS-7 cells were a gift from Dr. Cathy Carline, Case Western Reserve University, Cleveland, OH. MEM and DMEM supplemented with 10% heat-inactivated fetal bovine serum, penicillin/streptomycin, and 1% L-glutamine (Life Technologies, Gaithersburg, MD) were used to culture MDA-MB 231 cells, HeLa cells and COS-7 cells, respectively. All cells were maintained at 37°C in humidified tissue culture incubator with 6% CO2. All experiments were performed on cells maintained for a maximum of 10–15 passages. Specific inhibitors used are ML-7, Blebbistatin and Y-27632 purchased from Calbiochem (La Jolla, CA). Cell dissociation reagent and fibronectin were purchased from Sigma, St. Louis, MO. siRNA oligonucleotides specific to nonmuscle myosin IIA and IIB were purchased from Dharmacon RNA Technologies, USA. Transfection reagents were purchased from Amaxa Biosystems, USA. Alexa Fluor® 568 Phalloidin and DAPI nucleic acid stain and Alexa-conjugated secondary antibodies were from Molecular Probes, Eugene, OR. SuperFemto Western blot reagents were from Pierce, Rockford, IL. Antibodies purchased from Sigma (St. Louis, MO) include nonmuscle myosin IIA, myosin IIB, vinculin and actin. Paxillin and zyxin antibodies were from Zymed Laboratories Inc., San Francisco, CA. Focal adhesion kinase (FAK) antibodies were purchased from Santa Cruz Biotechnology, Santa Cruz, CA.

### Plasmids

To construct pDsRed-myosin IIA-ACD plasmid vector expressing myosin IIA assembly competence domains, a PCR product amplified by using 5′-GGAAGATCTAACACCGACCTGAACCTGGAGCGC-3′ and 5′-CGCGGATCCTTAGGGCACGACAAACGGCAGGTC-3′ primers from pEGFP-myosin IIA plasmid as template was cloned in pDsRed2-Nuc expression vector (BD Biosciences, San Jose, CA) as BglII and BamH1 fragment and thus deleted nuclear localization signal. Similarly, a PCR product amplified from pEGFP-myosin IIB by using 5′-GGAAGATCTGATGAGAAGCGGCGTCTGGAAGC-3′ and 5′-CGCGGATCCTTAGGGGCCACCCCGCCTCAGCCG-3′ was cloned as BglII and BamH1 fragment in pDsRed vector to generate pDsRed-myosin IIB-ACD expression vector. The plasmid pEGFP-nonmuscle myosin IIA-C3 expresses the full length corrected human nonmuscle myosin heavy chain under the control of a constitutive CMV promoter, with EGFP fused at the amino terminus of the myosin IIA coding region. This plasmid, a gift from Dr. Anne Bresnick [20], [37], [38], was used to express wild type GFP-myosin IIA. A GFP-nonmuscle myosin IIB expression vector was constructed by insertion of the full length corrected nonmuscle myosin IIB into Age I/Dra III sites the pCMV-EGFP-C3 vector [39]. The construction of pmCherry-myosin IIA expression vector was described earlier [38].

### Cell Spreading and Adhesion Assays

Quantitative cell spreading assays to compare cell membrane extensions were carried out as described earlier [36]. HeLa-Clontech and COS-7 cells transiently expressing either GFP-myosin IIA or GFP-myosin IIB or GFP for 36–48 hours were used to perform spreading assays. Cells were allowed to spread for 60 min in the incubator and then stopped by directly adding fixing buffer followed by washing with phosphate buffered saline (PBS). Images of the spreading cells were collected using inverted light microscope. To study cell adhesion efficiencies, cultured or transiently expressing cells (24–72 hours after transfections) were collected using cell dissociating reagent. Equal number of cells were seeded on fibronectin (20 µg/ml) coated 48-well tissue culture plates in growth medium. After incubating at 37°C for 60–70 min, growth medium was removed and cells were washed with PBS. Cells were fixed using paraformaldehyde (4%) as described earlier [36]. Nuclei of cells were stained with 4′, 6-diamidino-2-phenylindole (DAPI) and images of the attached cells were collected using microscope. Cells were counted using ImageJ program (NIH). To study adhesion efficiency in the presence of myosin II inhibitors, MDA-MB 231 collected from tissue culture plates were incubated with ML-7 (5 µM), blebbistatin (100 µM) and Y-27632 (5 µM) for 10–15 min before seeding on fibronectin coated plates.

### Immunostaining, Imaging and Western Blot Analysis

Preparation of total cell lysates, western blot analysis, immunostaining and imaging of the spreading cells were performed as described earlier [36]. For western blot analysis, total lysates were made from the spreading cells. Cells transiently expressing GFP-myosin II motor proteins and GFP for 36–48 hours were used for making total lysates.

## Results

### Myosin IIA Negates IIB-Mediated Lamellipodia Extension during Spreading

In earlier siRNA knockdown studies in MDA-MB 231 breast cancer cells, we observed distinct and contrasting effects on lamellipodia extension when myosin IIA versus IIB was subjected to siRNA depletion [36]. In those studies, depletion of myosin IIB reduced lamellipodia extension on fibronectin surfaces, implying a positive role for this ubiquitously expressed motor protein in generating cell membrane protrusion. Surprisingly, depletion of another abundant motor protein myosin IIA enhanced lamellipodia extension, suggesting that the contractile forces and tension generated by myosin IIA are not favorable to leading edge protrusion in this setting. This argues against the prevailing hypothesis that myosin II-mediated contractile forces are limited to retrograde actin flow in the lamellipodium [5], [7], [40]. It is often necessary to confirm these unexpected results by an alternative approach as certain siRNAs are known to display off-target effects, poor sub-cellular distribution, induction of interferon response, and silencing chromatin [41]–[45].

**Figure 1:**
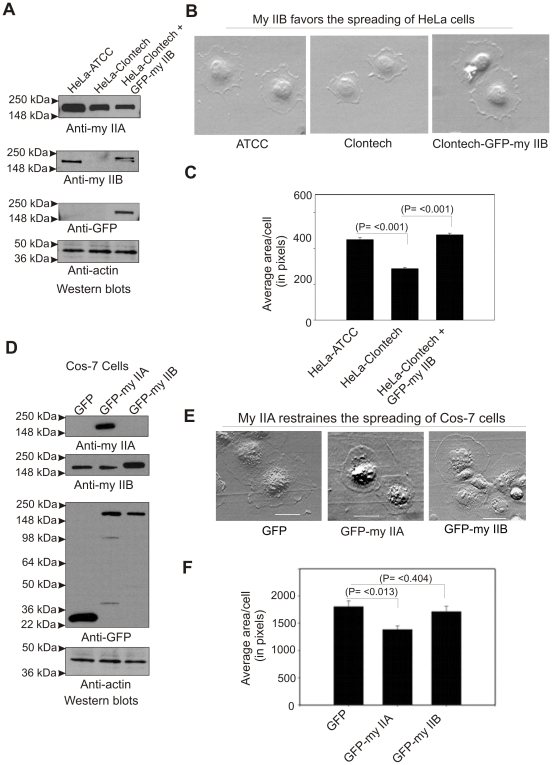
Myosin IIA and IIB mediate cell membrane extension in opposite directions. A) Western blots showing the expression of myosin II isoforms in HeLa cells. B) Myosin IIB favors lamellipodium extension during cell spreading. C) Graphical representation of the cell spread areas. Cells spread for 60 min were stopped by directly adding fixing buffer followed by staining with Alexa-Fluor conjugated wheat germ agglutinin to enhance cell margin contrast, and quantifying cell area using the ImageJ program (NIH) as described in Methods. For each presented bar, n = 350–450 cells quantified. The average spread area of HeLa-ATCC cell was considered as 100% to calculate the spread area of other cells. D) Western blots showing the expression of myosin II isoforms in COS-7 cells. E) Myosin IIA mediates lamellipodia retraction during cell spreading. F) Graphical representation of the cell spread areas. For each presented bar, n = 550–650 cells quantified. Bars represent standard errors, t-test. The average spread area of GFP alone expressing cell was considered as 100% to calculate the spread area of other cells.

As noted earlier [46], HeLa cells (HeLa-ATCC) obtained from The American Type Tissue Culture Collection, Manassas, VA express both myosin IIA and IIB isoforms. In contrast, the derivative tetracycline regulated HeLa cell line (HeLa-Clontech) generated by Clontech (Mountain View, CA) express only myosin IIA, but not IIB (Fig. 1*A*). Spreading assays performed on fibronectin coated surfaces demonstrated a significant impairment in the formation of lamellipodia extensions (≥36% reduction in the spread area of a cell) by HeLa-Clontech cells compared to HeLa-ATCC cells. Transient expression of GFP-myosin IIB corrected the impairment of lamellipodia formation by HeLa-Clontech cells (Fig. 1*B* and 1*C*). These results demonstrate that myosin IIB favors the extension of the cell membrane during spreading. This conclusion is further supported by similar analysis performed with COS-7 cells. COS-7 cells express myosin IIB, but have no detectable levels of myosin IIA (Fig. 1*D*). Transfection of COS-7 cells with GFP-myosin IIA reduced lamella spreading relative to control GFP-transfected cells (≥24% reduction in spread area (Fig. 1*E* and 1*F*). Collectively, these results support the hypothesis that 1) myosin IIA predominantly confers contractile and retrograde force at the cell margin, 2) myosin IIB predominantly confers protrusive force at the cell margin and 3) both myosin IIA and IIB contribute to overall lamellipodia extension on extracellular matrix. However, further studies are necessary to understand how myosin IIA and myosin IIB are involved in driving lamellipodia extension in opposite directions during cell spreading.

### Both Myosin II Isoforms Undergo Assembly Regulation during the Extension of Lamellipodia

**Figure 2:**
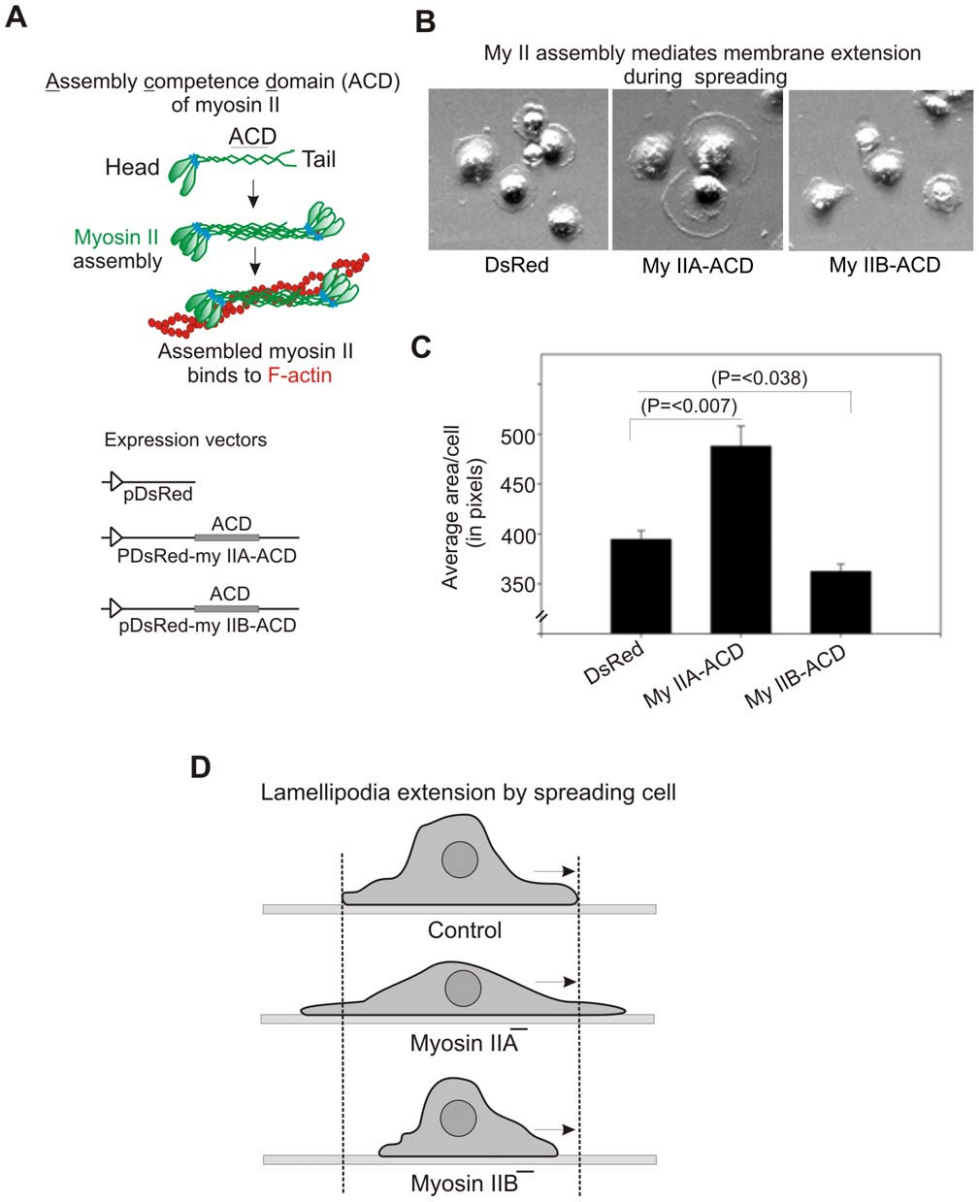
Myosin II undergoes assembly regulation during cell spreading. A) Myosin II assembly and construction of myosin II-ACD expression vectors. MDA-MB 231 cells were used for transient expression of myosin II-ACD. B) Spreading MDA-MB 231 cells transiently expressing myosin II-ACD. Images were collected after 60 min spread. C) Graphical representation of spreading areas of cells expressing myosin II-ACD. Spread areas of cells were measured as described under Fig. 1 legend. For each presented bar, n = 350–400 cells quantified. Bars represent standard errors, t-test. D) A schematic diagram showing the opposite roles of myosin IIA and IIB in extending lamellipodia during spreading.

Myosin II motor proteins are believed to be involved in mediating cellular functions by undergoing filament assembly in the cells. Overexpression of myosin II carboxyl terminal tail domains (“assembly competence domain“ or ACD) has been used in earlier work to interfere with normal myosin filament assembly in the cells [47]–[50]. To test whether myosin II filament assembly is important for displaying isoform specific roles of myosin IIA and IIB during lamellipodia extension, plasmid DNA vectors were created to transiently express red fluorescent protein (RFP) fusions of myosin II-ACD domains (Fig. 2*A*). Upon spreading, a significant enhancement in cell membrane extensions (≥14% increase in spread area) was observed in cells transiently expressing the RFP-myosin IIA-ACD fusion protein in comparison with cells expressing RFP only. In contrast, an impairment of lamellipodia extension (≥12% decrease in spread area) was observed in cells expressing RFP-myosin IIB-ACD (Fig. 2*B* and 2*C*). These results support the hypothesis that myosin IIA and IIB exert opposing effects on lamellipodia extension by undergoing filament assembly during cell spreading (Fig. 2*D*). If both myosin II motor proteins are involved in regulating lamellipodia extension in opposite direction by undergoing assembly regulation, it is likely that their sub-cellular localizations in spreading cells are different.

### Distinct Localizations of Myosin IIA and IIB in the Spreading Margins

**Figure 3:**
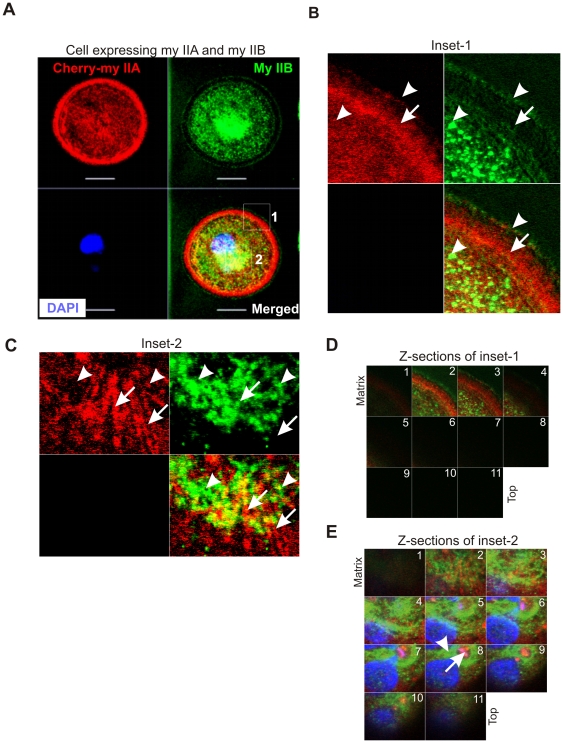
Distinct localizations of myosin IIA and IIB in spreading MDA-MB 231 breast cancer cells. A) Spreading cell transiently expressing cherry-myosin IIA was fixed and stained with myosin IIB antibody. A series of Z-sections were collected using confocal microscope. Inset-1 and inset-2 were drawn to study colocalizations of myosin IIA and IIB at the spreading margins and in the cytoplasm, respectively. B) and C) Blowup of a z-sections collected near the matrix. Arrow indicates cherry-myosin IIA and arrow-head shows endogenous myosin IIB. D) and E) Myosin IIA and IIB display distinct localization in the spreading margins and cytoplasm. A series of z-sections were collected using confocal microscope.

To understand their specific localizations in the spreading margins, MDA-MB 231 breast cancer cells were transfected with a Cherry-myosin IIA expression vector, fixed during active spreading on fibronectin coated surface, and immunostained to localize endogenous myosin IIB. Both isoforms were significantly enriched in the cell margins, but exhibited distinct localizations (Fig. 3*A* and 3*B*). Myosin IIB enrichment was observed towards the outer margin of the cell, with a fine punctate appearance. Myosin IIA was slightly further back in the lamellipodium. Notably, myosin IIB appears to be localized to punctuate structures both at the extreme margin and in the more central zones, an appearance not seen for myosin IIA (Fig. 3*B* and 3*C*). Their localization is different in the central zone that is away from the matrix. Myosin IIA appears less diffuse in comparison with IIB (Fig. 3*D* and 3*E*). Fluorescence recovery after photobleaching (FRAP) studies also showed a substantial difference in the recovery rates and mobility of GFP-tagged myosin IIA and IIB localized in the spreading margins of cells (Breckenridge and Egelhoff, unpublished). These studies combined with isoform rescue studies suggest that myosin IIA and IIB have distinct and opposite roles in leading edge mechanics and protrusion. However, further studies are essential to understand the mechanism by which both myosin IIA and IIB mediate lamellipodium extension in opposite directions during spreading.

### Myosin IIA and IIB with Separate, but Linked Roles, Contribute to Cell Attachment to Matrix during Spreading

**Figure 4:**
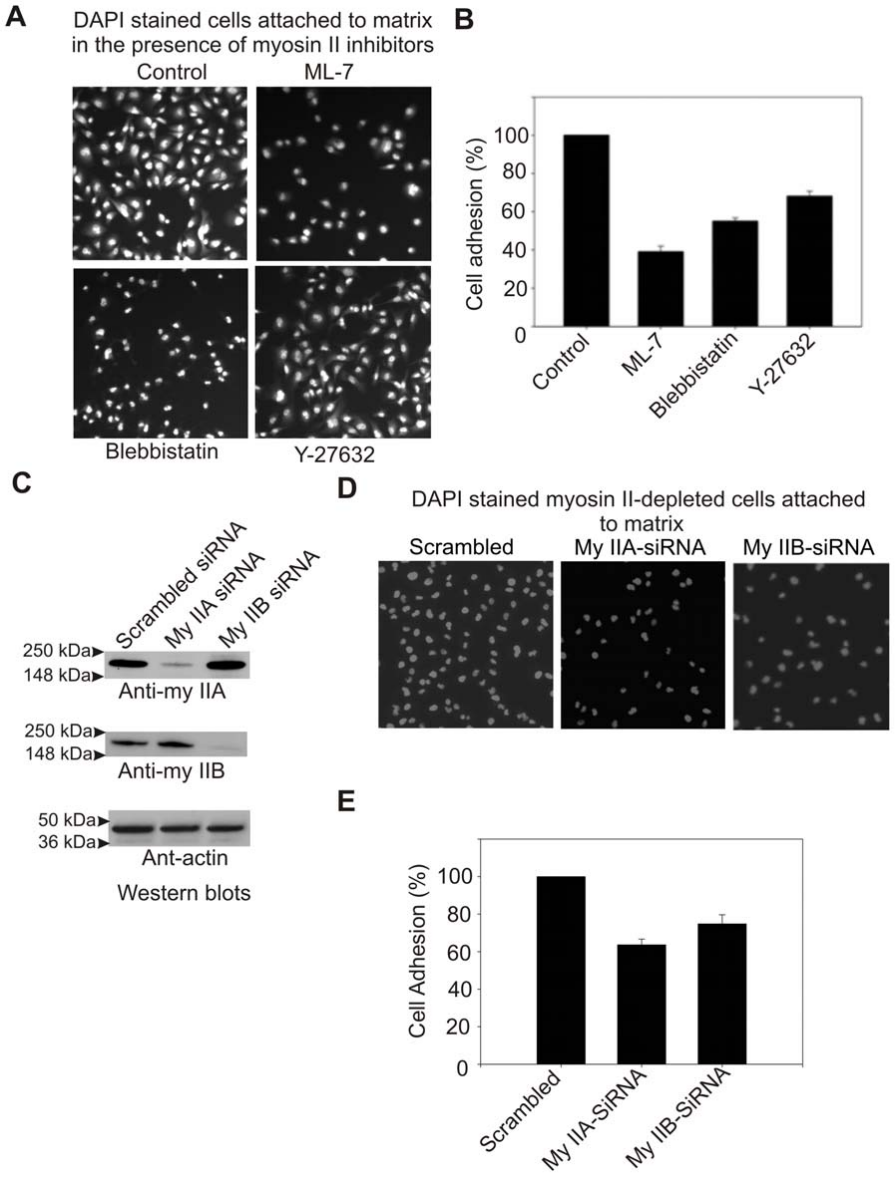
Myosin IIA and IIB involved in the regulation of cell attachment to matrix. A) Pharmacological inhibition of myosin II impairs cell attachment to matrix. B) Graphical representation of cell adhesion impairment in the presence of myosin II inhibitors. DAPI stained nuclei on the images were quantified using ImageJ program (NIH) as a measure of cell number. The average number of cells attached in the control population was set as 100%. C) Western blots showing depletion of myosin II isoforms in siRNA electrophorated cells. MDA-MB 231 cells were electrophorated with either a control scrambled siRNA, siRNA to myosin IIA, and myosin IIB. Total cell lysates made from cells grown for 72 hour were subjected to Western blots analysis. D) Myosin IIA and IIB are required for cell adhesion. E) Graphical representation of adhesion deficiency of myosin II depleted cells. The average number of cells attached in the control scrambled siRNA population was set as 100%. Columns mean (n = 3 for each treatment condition); bars, SE.

**Figure 5:**
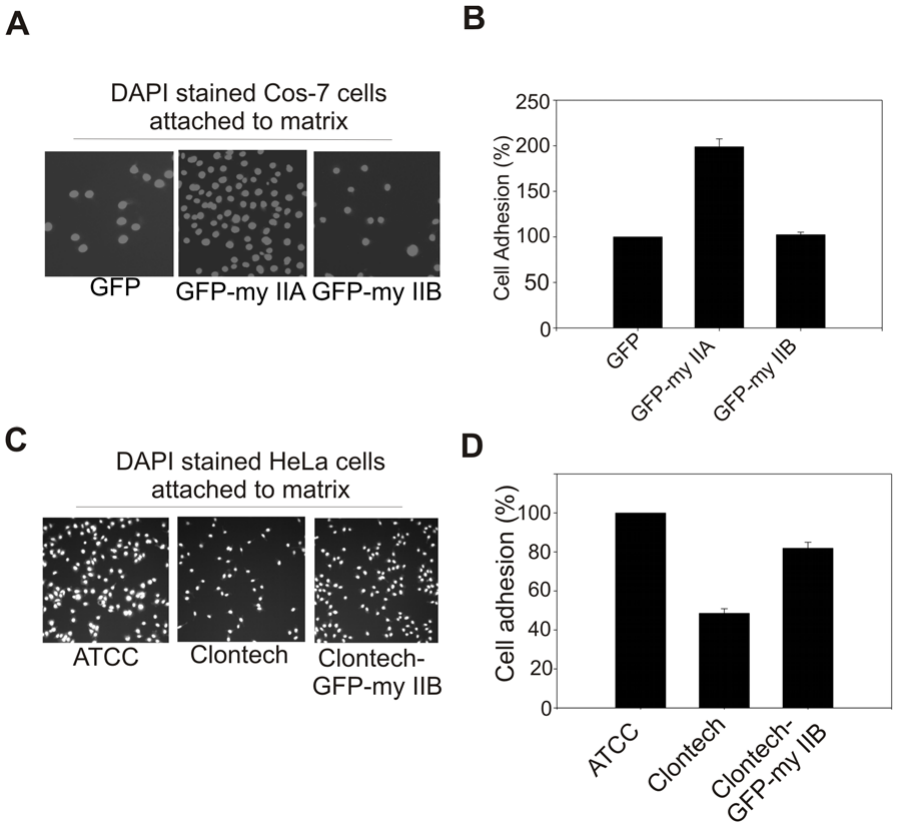
Both myosin IIA and IIB are required for the efficient cell attachment to matrix. A) Transient expression of GFP-myosin IIA increases Cos-7 cells attachment to fibronectin matrix. B) Graphical representation of Cos-7 cells attachments to matrix. C) Transient expression of GFP-myosin IIB increases HeLa-Clontech cells attachment to fibronectin matrix. D) Graphical representation of HeLa-Clontech cells attached matrix. The average number of COS-7 cells transiently expressing GFP and HeLa-ATCC cells attached to matrix were set as 100%. Columns mean (n = 3 for each condition); bars, SE.

During lamellipodia extension the cell membrane interacts with matrix by forming focal contacts (focal complexes, focal adhesions and fibrillar adhesions are collectively termed as focal contacts in the present manuscript). The cell membrane interaction with matrix has direct impact on lamellipodia extension during spreading and migration. Myosin II is believed to play critical roles in regulating membrane interaction with matrix [51], [52]. To understand isoform specific roles of myosin II in regulating cell membrane interaction with matrix, cell adhesion assays were performed using different cell lines and pharmacological inhibitors. Pharmacological inhibition of myosin II light chain kinase (MLCK), Rho-associated coiled-coil containing protein kinase (ROCK) and myosin II motor activity has shown a significant impairment of cell attachment (39–70% reduction, n = 900–1100 cells) to fibronectin coated surface (Fig. 4*A* and 4*B*). Cells depleted for each myosin II isoform were tested for adhesion during the active phase of spreading on fibronectin coated surface (Fig. 4*C*). Isoforms-specific depletion of either myosin IIA or IIB resulted in substantial reduction (31–39% reduction, P<0.001, n = 750–850 cells) in adhesion of MDA-MB-231 cells to fibronectin matrix (Fig. 4*D* and 4*E*). To recapitulate these results, cell adhesion assays were performed using myosin II null cells. COS-7 cells expressing no endogenous myosin IIA are poorly attached to matrix. However, cell attachment was substantially increased upon transient expression of GFP-myosin IIA. Transient expression of either GFP or GFP-myosin IIB does not increase in COS-7 cell attachment to matrix (Fig. 5*A* and 5*B*). On the other hand, the HeLa-ATCC cells expressing both myosin IIA and IIB showed efficient attachment (nearly 100% increase) to matrix in comparison with the attachment of HeLa-Clontech cells lacking endogenous expression of myosin IIB. Transient expression of GFP-myosin IIB in HeLa-Clontech cells increases attachment substantially (Fig. 5*C* and 5*D*). This analysis indicates that both isoforms, IIA and IIB, contribute to overall adhesion during the initial stages of lamellipodia engagement with extracellular matrix (ECM) during spreading. These results suggest that both myosin IIA and IIB isoform are also involved in the regulation of cell membrane attachment to matrix during spreading.

### Myosin IIA and IIB Target Different Focal Contacts during Cell Spreading

**Figure 6:**
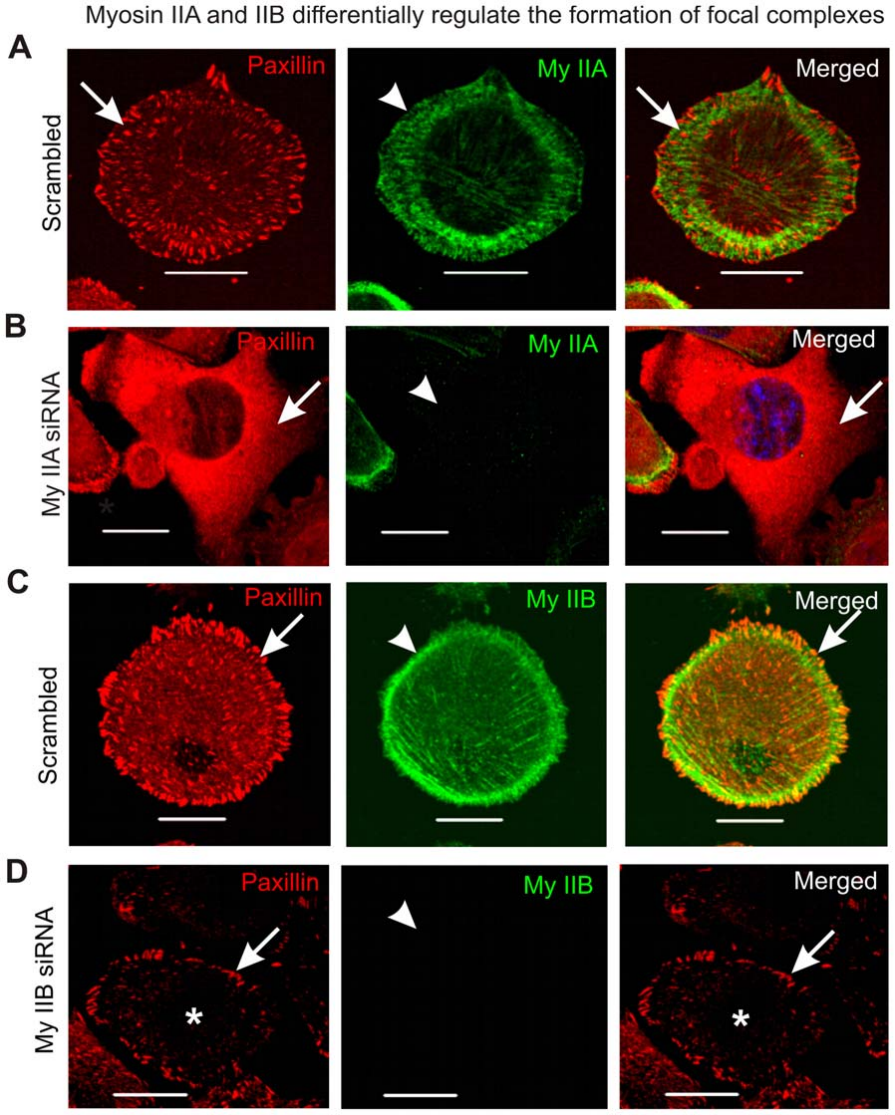
Myosin IIA and IIB display differential regulation of focal contacts during cell spreading. MDA-MB 231 cells collected after 72 hour of transfection with scrambled siRNA, myosin IIA siRNA and myosin IIB siRNA, were seeded on fibronectin coated surface and allowed to spread for 60 min in the incubator. Cells were fixed and stained with paxillin, myosin IIA and myosin IIB antibodies and DAPI as described in methods. A) Localization of myosin IIA and focal contacts in the spreading cell. Arrow and arrow-head indicate focal contact and myosin IIA, respectively. B) Loss of focal contacts formation in the cells depleted of myosin IIA. Arrow and arrow-head indicate loss of focal contacts and depletion of myosin IIA, respectively. C) Localization of paxillin and myosin IIB in the spreading cells. Arrow and arrow-head indicate paxillin stained focal contact and myosin IIB, respectively. D) Depletion of myosin IIB impairs formation of focal contacts in the central part of the spreading cell. Arrow and arrow-head indicate focal contacts formation in the cell margin and depletion of myosin IIB, respectively. Asterisk indicates loss of focal contacts formation in the central part of the spreading cell.

To better understand the isoform specific role in cell membrane interaction with matrix, MDA-MB 231 breast cancer cells actively engaged in extending lamellipodia were fixed and stained for focal contacts during spreading. Following siRNA depletion of myosin IIA or myosin IIB in MDA-MB 231 cells, western blot analysis was performed to assess expression of the major components of focal contacts. These studies revealed that depletion of either myosin IIA or IIB did not affect the total cellular abundance of paxillin, vinculin, focal adhesion kinase (FAK) or zyxin (Figure S1). Similar results were also observed in spreading cells lacking endogenous expression of myosin II isoforms (data not shown). These results indicate that myosin II isoforms contribute to cell attachment to matrix without affecting the overall abundance of some of the major components of focal contacts during spreading. However, these results do not rule out myosin II isoform specific roles in establishing focal contacts or regulating their behavior during spreading. To gain insights in the formation of focal contacts, spreading MDA-MB 231 cells were fixed and stained with paxillin antibody. These results show punctate, focal contact paxillin immunostaining throughout the basal membrane of the spreading cells expressing both myosin IIA and IIB (Fig. 6*A* and 6*C*). Few or none of these punctate focal contacts showed colocalization with myosin II. However, upon depletion of myosin IIA via siRNA, paxillin staining was substantially more diffuse, with a significant reduction in the number of cells displaying prominent focal contacts (Fig. 6*B*). Similar results were also observed in the cells spreading in the presence of ROCK inhibitor Y-27632 (data not shown). Upon depletion of myosin IIB via siRNA, focal contact abundance in the central regions of the actively spreading cells was reduced, although focal contacts along the cell edge were commonly observed in many cells (Fig. 6*D*). Inhibition of MLCK by using ML-7 has also revealed similar behavior of focal contacts during spreading (data not shown). These results indicate a specific role for myosin IIA in the formation of focal contacts especially in the spreading margins and myosin IIB role in the formation of focal contacts preferably in the central part of spreading cell.

### Transient Expression of Myosin IIA Rescues the Impairment in Focal Contact Formation in Cells Lacking Endogenous Protein

**Figure 7:**
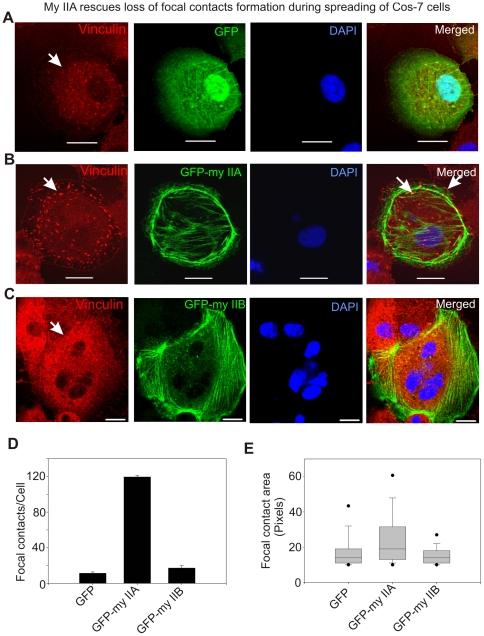
Transient expression of GFP-myosin IIA but not IIB rescues loss of focal contacts formation in spreading COS-7 cells. Cells were fixed after 60–70 min of spreading and stained with vinculin antibodies and DAPI. A) Loss of focal contacts in spreading COS-7 cells transiently expressing GFP. Arrow indicates loss of focal contact formation in the spreading cell. B) Transient expression of GFP-myosin IIA rescues formation of focal contacts. Arrow indicates focal contact formation. C) Transient expression of GFP-myosin IIB does not rescue focal contacts formation in the spreading cells. Arrow indicates loss of focal contact formation. D) Graphical representation of number of punctate structures (focal contacts) per cell. Confocal z-sections were exported into TIF images and then processed to quantify number and area of punctate structures using ImageJ program (NIH). Numbers of images processed to quantify number and area of punctate structures were in between 12–15. E) Box-and-whisker plot showing the average area of focal contact. We have observed a wide range of areas of punctate structures in pixels and therefore showed by Box-and-whisker plot.

The distinct effects of myosin IIA and IIB on the establishment or regulation of focal contacts could be due to off-target effects of siRNAs used in the above experiments. Therefore, COS-7 cells lacking endogenous expression of myosin IIA (Fig. 1*D*) were used to confirm the above effect on the establishment of focal contacts during spreading. COS-7 cells were transiently transfected with plasmid vectors expressing either GFP or GFP-myosin IIA. After 48 hour, cells were collected, allowed to spread for an hour on fibronectin-coated surface, then fixed and immunostained for vinculin. Although some focal contact structures could be detected in the center of COS-7 cells expressing GFP, but the spreading margins show no focal contacts (Fig. 7*A*). The loss of focal contacts formation in the spreading margins was completely rescued by transient expression of GFP-myosin IIA in COS-7 cells (Fig. 7*B*). Interestingly, no significant change in the formation of focal contacts was observed upon expression of GFP-myosin IIB in COS-7 cells (Fig. 7*C*). Further analysis of these focal contacts has revealed a significant increase in the average number of focal contacts formed by a spreading cell expressing GFP-myosin IIA in comparison with cells expressing GFP only (Fig. 7*C*). In addition, the average area of a focal contact was significantly increased upon transient expression of GFP-myosin IIA in the spreading COS-7 cells (Fig. 7*D*). Taken together, these results suggest a specific role for myosin IIA in the formation of matured focal contacts in the cells actively engaged in extending lamellipodia. These results also suggest that myosin IIA does not have a role in the nascent or initial focal contacts establishment or formation during spreading.

### Myosin IIB Regulates Focal Contacts Formation in the Central, but Not in the Lamellipodia during Cell Spreading

**Figure 8:**
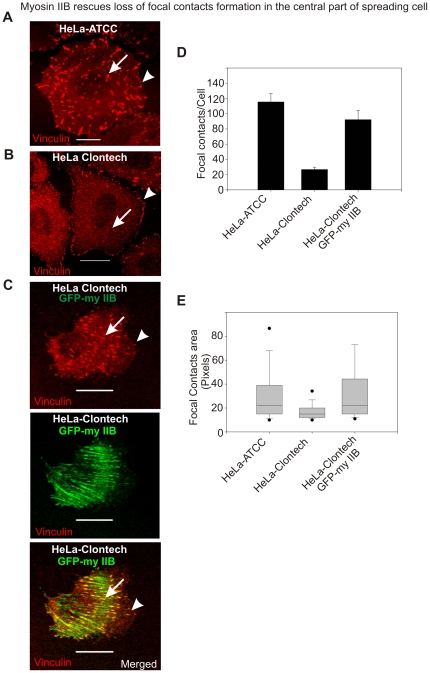
Myosin IIB is required for focal contacts formation in the central part of the spreading cells. A) Spreading HeLa-ATCC cell displaying focal contacts formation through out the membrane. Arrow and arrow-head indicate focal contacts formation in the central and cell margins of the spreading cell, respectively. B) Spreading HeLa-Clontech cells show focal contacts formation exclusively in the peripheral region. Arrow indicates loss of focal contacts formation in the central region and arrow-head shows the formation of focal contacts in the spreading margins. C) Transient expression of GFP-myosin IIB in HeLa-Clontech cells rescues the formation of focal contacts in the central part of spreading cell. The scale bars represent 20 µm. D) Graphical representation of focal contacts in spreading HeLa cells. E) Box-and-whisker plot showing the average area of focal contact. Numbers of images processed to quantify number and area of punctate structures were in between 12–16.

HeLa-ATCC cells expressing both myosin IIA and IIB (Fig. 1*A*) have typically displayed the formation of robust focal contacts during active spreading (Fig. 8A). While HeLa-Clontech cells lacking myosin IIB but express IIA, formed distinct marginal focal contacts during spreading, with reduced number of larger central focal contacts (Fig. 8*B* and 8*D*) compared to the HeLa-ATTC cells. Transient expression of GFP-myosin IIB into HeLa-Clontech cells caused a substantial increase not only in the number of focal contacts formed per cell, but also in the average area of each focal contact (Fig. 8*C*, 8*D* and 8*E*). This suggests that myosin IIB is involved in the regulation of focal contacts formation in the central part of spreading cell. However, further studies are essential to understand how myosin IIA and IIB exert their specific roles in regulating different focal contacts formation during cell spreading.

### Different Roles of Myosin IIA and IIB in Regulating the Actin Network in the Spreading Cells

**Figure 9:**
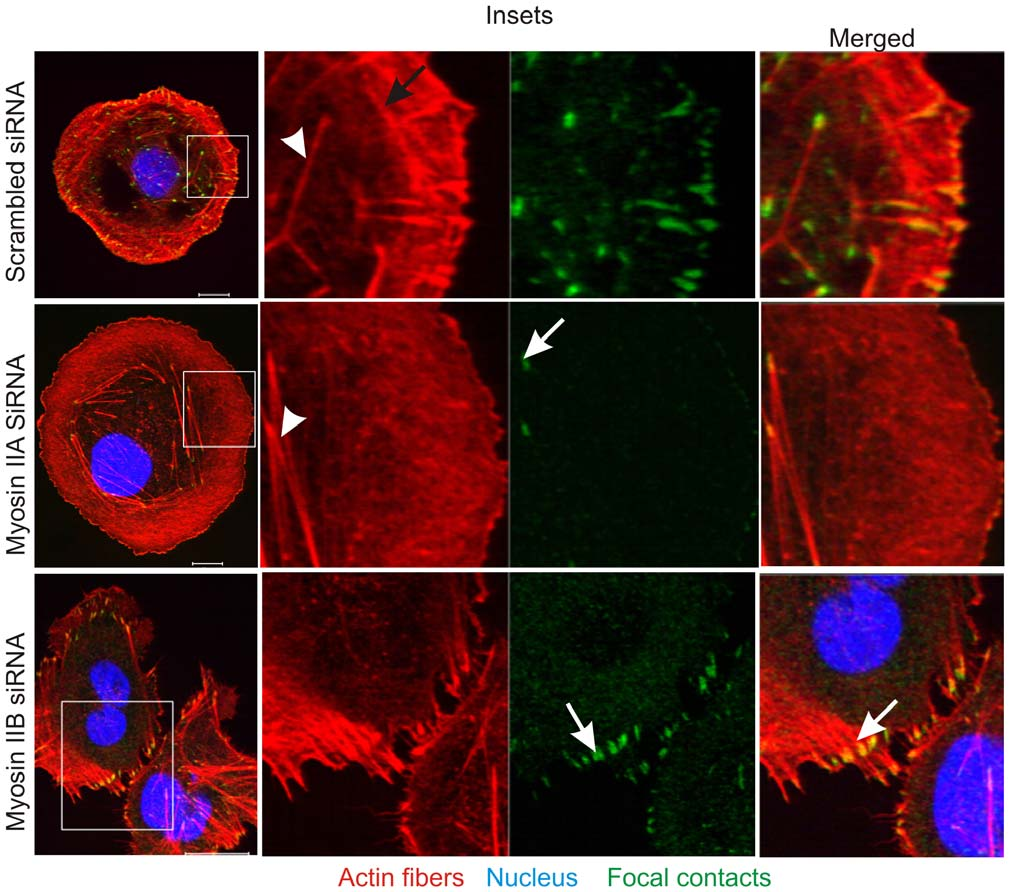
Both myosin IIA and IIB isoforms are required for the formation of distinct actin network in the spreading cells. Spreading MDA-MB 231 cells depleted of myosin IIA and IIB were fixed and stained with paxillin antibodies, phalloidin, and DAPI. A) The formation of an organized actin network in the spreading cell expressing myosin IIA and IIB. Arrow-head and filled arrow indicate central fibers and transverse arcs in the scrambled siRNA received spreading cells, respectively. Focal contacts are connected to actin filaments in the lamellipodia and central part of the spreading cell. Loss of an organized actin network especially actin arcs and focal contacts are shown in the spreading cell depleted of myosin IIA. Myosin IIB-depleted cells display loss of transverse arcs and the formation of long actin fibers during spreading. In the spreading margins focal contacts are connected to these long actin filaments. The scale bars represent 20 µm.

Recent studies showed reorganization of the actin network and its roles in the formation of focal contacts and lamellipodia extension during cell spreading [53]. Impairment of the actin-bundle turnover was demonstrated in neuronal growth-cones upon pharmacological inhibition of myosin II motor activity [6]. It is likely that myosin IIA and IIB motor proteins regulate the actin network reorganization differently to exert isoform specific roles in the formation of focal contacts and lamellipodia during cell spreading. To gain insights in the mechanisms by which myosin IIA and IIB isoforms mediate the actin network reorganization process, the spreading cells were analyzed for actin network and focal contacts using myosin II-depleted cells. These studies revealed the formation of an organized actin network that includes retrograde flow actin filaments in the front followed by a distinct actin arcs structures in the spreading margins of MDA-MB 231 cells expressing both myosin IIA and IIB motor proteins (Fig. 9). The actin arcs structures are parallel to each other and cell's edge and are located in the back of lamellipodium. Some of the filaments originated from arcs are connected to focal contacts. Beyond this distinct band of 2–3 µm transverse actin arcs, another set of actin network comprising parallel actin filaments is present in the center of the spreading cell (arrow head). These central actin filaments are perpendicular to the actin arc structures located in the lamellipodium, but are parallel to retrograde actin filaments present in the spreading margins. No such organized actin network is observed in the spreading cells depleted of myosin IIA. Myosin IIA depleted cells, but expressing IIB, display loss of distinct actin arc structures with a few central actin filaments and appear to have fragmented actin network diffused especially in the spreading margins. Although a few focal contacts connected to central filaments are present in the center of the spreading cell, but the spreading margins show no focal contacts. The spreading cells depleted of myosin IIB, but expressing IIA, display straight long and parallel actin filaments across the cytoplasm with no actin arcs in the spreading margins (Fig. 9). These elongated actin filaments are connected to focal contacts in the spreading margins of myosin IIB-depleted cells. These results suggest that both myosin IIA and IIB are essential to form actin arcs in the spreading margins. These results also suggest that both myosin IIA and IIB involve in the formation of an organized actin network that is critical to extend lamellipodia and membrane interaction with matrix during cell spreading.

## Discussion

Lamellipodium is a characteristic feature at the leading edge and is believed to be the actual motor and steering device that maneuvers cell during migration. The present study demonstrates an unexpected opposite mechanical roles for myosin IIA and IIB motor proteins in mediating lamellipodium extension during spreading. Both motor proteins involve in determining the fate of lamellipodia extension by displaying distinct, but linked roles in the regulation of focal contacts formation and actin network reorganization. In that view, the underlying mechanism of lamellipodia extension appears more complex than previously thought in higher organisms as the role of myosin II is confined to the establishment of cell polarity during migration of lower organisms like *Dictyostelium discoideum* that carries a single copy of myosin II gene [54]. The evidence presented in the current study initiates debate on how higher organisms use multiple myosin II motor proteins to drive cell migration and other cellular processes. Involvement of myosin IIA and IIB in the mediation of different cellular functions strongly support the hypothesis of isoform specific with little or no redundant roles in driving lamellipodia extension proposed in the present study [22], [24], [26], [36], [55]–[59].

Conventional models depict that lamellipodium extension occurs as a result of protrusive forces generated by the formation of F-actin from Arp2/3 mediated G-actin polymerization and other cellular processes like exocytosis at the plasma membrane [2]–[4], [60], [61]. Interestingly, migrating cells prefer to make periodic lamellipodia extensions by inducing F-actin reorganization and or retrograde flow in the lamellipodium. The role of myosin II that is known to associate with F-actin is implicated in mediating lamellipodium retraction using chemical inhibitors during cell spreading and directed migration [5]. In other words, inhibition of myosin II activity should favor lamellipodia extension. This raises an intriguing question which myosin II isoform is responsible for retraction and or attenuation of lamellipodia extension during spreading or migration. The present study provides evidence for myosin IIA, but not myosin IIB involvement in opposing the extension of lamellipodia using COS-7 cells that lack endogenous expression of myosin IIA (Fig. 1*E* and 1*F*). Previous work shows a significant assembly of myosin IIB in the lamellipodia implying an overlapping function with myosin IIA during cell spreading [36]. Using HeLa-Clontech cells that lack endogenous expression of myosin IIB, the present study clearly demonstrates a positive mechanical role for myosin IIB in driving lamellipodia extension (Fig. 1*B* and 1*C*). Interestingly, myosin IIB contributes for the cell membrane extension by displaying little or no colocalization with myosin IIA in the lamellipodia of the spreading MDA-MB 231 human breast cancer cells (Fig. 3) that is akin to earlier observations made in growth cones [62]. These results together with a significant increase in retrograde flow of F-actin observed in growth cones of myosin IIB knockout mice [63] support the notion that myosin IIA and IIB play separate and opposite mechanical roles in mediating lamellipodia extension during cell migration. Mammalian cells expressing myosin IIA and IIB motor proteins might have opted such a mechanism to probe the environment and to prevent accumulation and bundling of F-actin at the back of lamellipodium due to retrograde flow [5]. From earlier studies implicating in cell migration promoting exocytosis and punctate appearance in the cell's edge shown in the present study, the role of myosin IIB in driving exocytosis to promote lamellipodium formation cannot be ruled out. However, further studies are needed to understand the role of myosin IIB in extending lamellipodia during cell migration.

**Figure 10:**
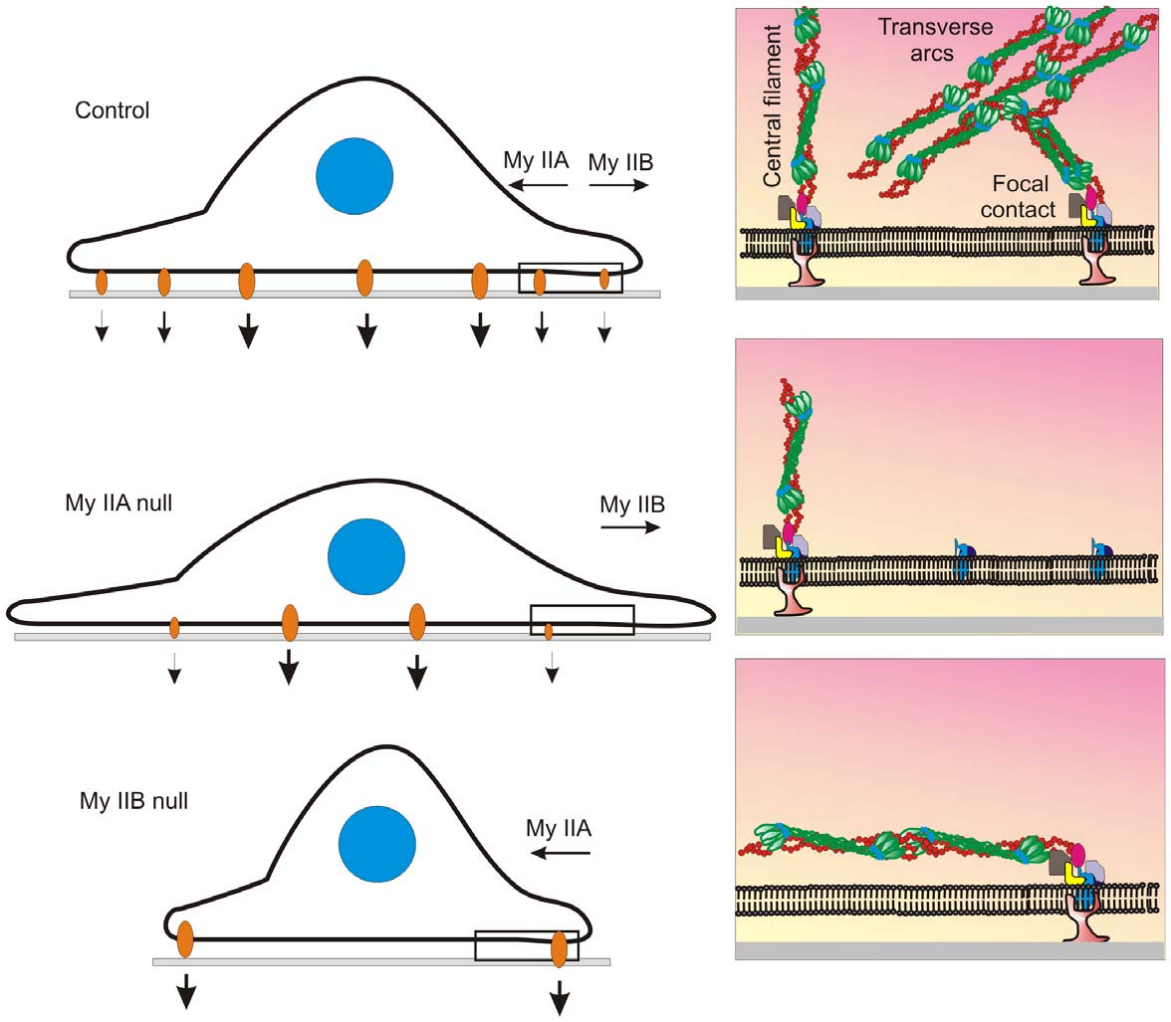
Schematic diagrams depicting opposite but linked mechanical roles for myosin IIA and IIB in determining the fate of lamellipodial extension during cell spreading.

Recent studies reveal localization of actin arcs in the back of lamellipodium called transition zone or lamella. Actin arcs comprise actin filaments and bundles that are oriented in the direction of cell edge and perpendicular to retrograde F-actin in the lamellipodium [6]. The role of myosin II has been implicated in regulating the reorganization of actin network in the lamellipodia of the spreading cells [6], [8]. The present study reveals the formation of an unorganized actin network with no distinct F-actin, actin arcs, and central stress fibers in the spreading cells expressing only myosin IIB but not myosin IIA (Fig. 9). This suggests positive roles for myosin IIA in establishing or forming actin arcs in the spreading cells. Conversely, spreading cells depleted of myosin IIB displayed long and parallel actin filaments in the cytoplasm with no actin arcs and central fibers (Fig. 9). This suggests independent but linked roles for myosin IIA and IIB in the reorganization of the actin network in the spreading cells. Myosin II roles in the regulation of focal contacts are known [64], [65], but isoform specific functions are not clearly understood. The present study reveals no significant effect on the formation of the initial or nascent focal contacts, but demonstrated distinct role for myosin IIA in the formation of stable or mature focal contacts especially in the spreading margins (Fig. 6 and 7). In contrast, myosin IIB has a role in the regulation of focal contacts that are limited to the central part, but not to the margin of the spreading cell (Fig. 6 and 8). As focal contacts regulate the cell membrane and matrix interaction, the impaired membrane interaction with matrix due to loss of focal contacts could be responsible for a significant increase in lamellipodia extension observed in myosin IIA null cells during spreading (Fig.1*D, E* and *F*). Myosin IIB null cells show impairment in membrane protrusion (Fig. 1*A, B,* and *C*) because these cells still express myosin IIA and form mature focal contacts at the cell edge (Fig. 6 and 8) that stabilizes membrane interaction with matrix and impede membrane extension during spreading as summarized in Fig. 10.

Thus, the present study demonstrates that myosin IIA and IIB with separate but linked mechanical roles determine the fate of lamellipodia extension during cell spreading. It is also postulated that deregulation or differential expression of myosin II motor proteins may have impact on cell migration and development of pathological conditions. The opposite roles of myosin IIA and IIB could be responsible for reporting many conflicting results in myosin II-mediated cellular processes. Expression of more than a single myosin II motor protein offers an extra leverage to have a tight control on cellular processes in higher organisms. However, further studies are essential to unravel the complex mechanisms involved in the reorganization of actin network mediated by myosin IIA and IIB during cell spreading and migration.

## Supporting Information

Figure S1 Depletion of myosin II isoforms display no effect on the expression levels of the components of focal contacts during cell spreading.
(3.46 MB TIF)
